# Supplementary material for: Effects of pay rate and instructions on attrition in crowdsourcing research
Source: PLoS One. 2023 Oct 4;18(10):e0292372. doi: 10.1371/journal.pone.0292372 (PMC10550147; doi:10.1371/journal.pone.0292372)
Supplement: S1 Appendix — (DOCX) [file pone.0292372.s001.docx]

**S1 Appendix**

**Task Description**

For both tasks, participants made responses on their keyboard as one (go/no-go reversal task) or two circles with distinctive patterns (two-choice reversal task) appeared on the computer screen. In each task, patterns were randomly selected from a set of six stimuli – see top panel of Figure S1, included with supplemental materials. Each task consisted of three trial blocks with two of the six stimuli appearing either simultaneously or successively across trials within each block. There was a 5-s “blackout” between each trial block, consisting of the removal of onscreen stimuli and presentation of a white hourglass image atop a black background.

The following instructions appeared in the app before the start of the first task:

This is the first of two tasks. In each task, try to earn as many points as possible. If you see a green smiley face, you earned one point. If you see a red frowny face, you did not earn a point.

At the onset of each task, a white fixation cross appeared at center of screen for 800 ms, and again for the same duration after each trial. Feedback on each trial consisted of a green smiley face for “correct” responses or red frowny face for “incorrect” responses. The probability of each of these consequences was .9 on each trial; the probability of receiving the opposite feedback (e.g., red frowny face for a “correct” response) was .1 on each trial. Total points earned appeared on the screen upon completion of each task.

The following instructions appeared before the two-choice reversal task:

Choose a pattern to earn points in this task. You will have to work out which pattern to choose. If you want to select the pattern on the left, press the 'E' key. If you want to select the pattern on the right, press the 'I' key. Press the spacebar to begin.

The bottom panel of Figure S1 shows an example of the task interface. On each trial, two patterns simultaneously appeared in circles that were vertically centered on the screen, with the diameter of each circle corresponding to 30% of the screen’s height. The arrangement of patterns (left or right side of the screen) was randomly selected on each trial. Pressing ‘E’ or ‘I’ resulted in a red arrow appearing above the pattern on the left or right side of the screen, respectively. At the same time, feedback was provided – that is, a green smiley face or red frowny face appeared between the two patterns. If participants did not make a response within 1200 ms, red text appeared (“Too Late!”) centered above the two patterns. Each trial block consisted of 40 trials. On Trial 21 of each trial block, the “correct” and “incorrect” patterns reversed without warning. The task ended upon completion of the third trial block (120 trials).

The following instructions appeared before the successive reversal task:

To earn points in this task, PRESS THE SPACEBAR as quickly as possible when a pattern appears *OR* DO NOT RESPOND AT ALL. You will have to work out when to respond or not respond. Press the spacebar to begin.

On each trial, one pattern appeared in a circle that was vertically centered on the screen, with the diameter corresponding to 30% of the screen’s height. One pattern was the “go” stimulus and a second pattern was the “no-go” stimulus. Pressing the spacebar (go stimulus) or withholding a response (no-go stimulus) resulted in removal of the stimulus and delivery of feedback. If the participant did not make a response, feedback was provided after 1200 ms. Each trial block consisted of 80 trials such that each pattern was presented 40 times within a trial block. On Trial 41 of each trial block, the “go” and “no-go” stimuli reversed without warning. The task ended upon completion of the third trial block (240 trials).

**Instructions on Prolific Page**

Part 1

Title: Play a game for academic research (Part 1)

Study overview:

Part 1: play a game for academic research.

Part 2: complete a survey about your participation.

*****THIS IS A TWO-PART STUDY. YOU WILL GET PAID ONLY AFTER COMPLETING PART 2.*****Most participants complete both parts in ~ 30 min.

Enter the completion code provided at the end of the game to confirm that you have completed part 1. Then, you will be invited to complete part 2.

**Please note that this study will ask you to download the free Inquisit Web app. Inquisit Web runs on Firefox, Chrome, Internet Explorer, and Safari.**

Part 2

Title: Play a game for academic research (Part 2)

This is part 2 of the study. You will complete a survey.

*Note: Estimated task duration, estimated pay rate, and base pay amount were also displayed on the Prolific page.

**Table S1**

Participant Demographics (N = 152)

| Measure | *M* (*SD)* | *n* |  | Percent of Sample |
| --- | --- | --- | --- | --- |
| Age | 31.5 (10.8) |  |  |  |
| Autism-Spectrum Quotient (AQ) Score | 20.4 (6.7) |  |  |  |
| Sex |  |  |  |  |
| Male |  | 83 |  | 54.6 |
| Female |  | 65 |  | 42.8 |
| Other |  | 2 |  | 1.3 |
| Not reported |  | 2 |  | 1.3 |
| Race/Ethnicity |  |  |  |  |
| White |  | 99 |  | 65.1 |
| Asian |  | 28 |  | 18.4 |
| Black/African American |  | 15 |  | 9.9 |
| Latinx |  | 5 |  | 3.3 |
| Hawaiian/Pacific Islander |  | 1 |  | 0.7 |
| Other |  | 2 |  | 1.3 |
| Not reported |  | 2 |  | 1.3 |
| Nationality |  |  |  |  |
| American |  | 137 |  | 90.1 |
| Other |  | 13 |  | 8.6 |
| Not reported |  | 2 |  | 1.3 |
| Country of Residence |  |  |  |  |
| United States |  | 151 |  | 99.3 |
| Not reported |  | 1 |  | 0.7 |
| Education |  |  |  |  |
| Some high school  High school diploma  Some college/vocational training  College degree  Professional or graduate degree  Not reported |  | 3  14  43  61  29  2 |  | 2.0  9.2  28.3  40.1  1.3  1.3 |
| Color blindness |  | 2 |  | 1.3 |
| Red-green |  | 2 |  | 1.3 |
| Not reported |  | 3 |  | 2.0 |
| Clinical Diagnoses |  |  |  |  |
| ASD |  | 1 |  | 0.7 |
| ADHD |  | 15 |  | 9.9 |
| ASD and ADHD |  | 3 |  | 2.0 |
| Other |  | 17 |  | 11.2 |

**Figure S1**

*Task Interface and Stimuli*

Set 1:


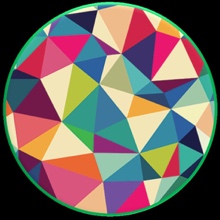

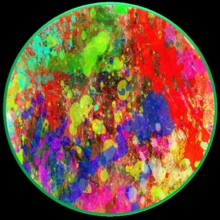

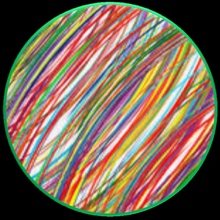

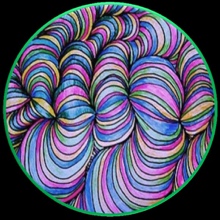

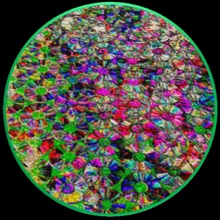

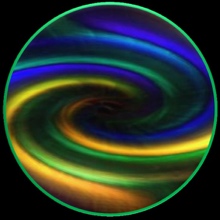


Set 2:


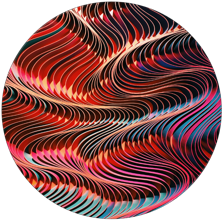

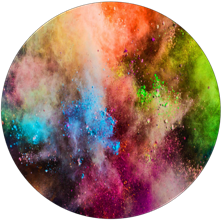

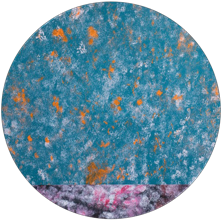

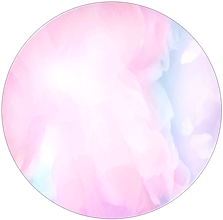

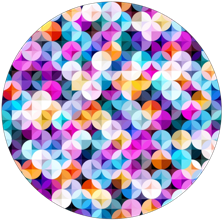

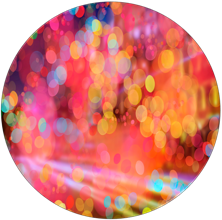


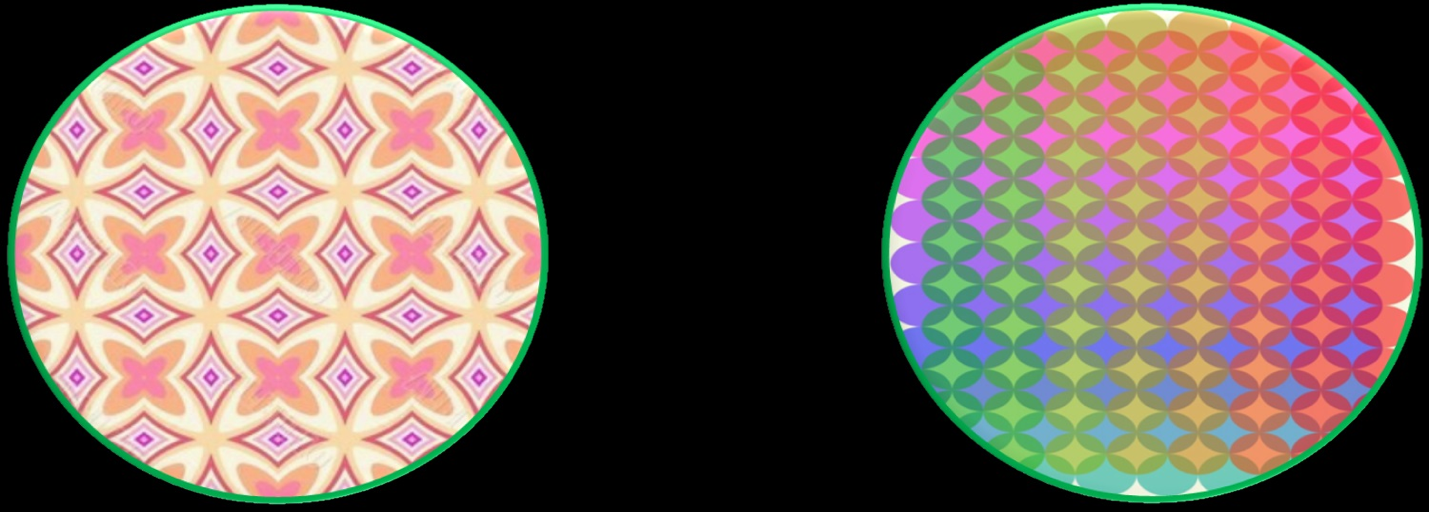

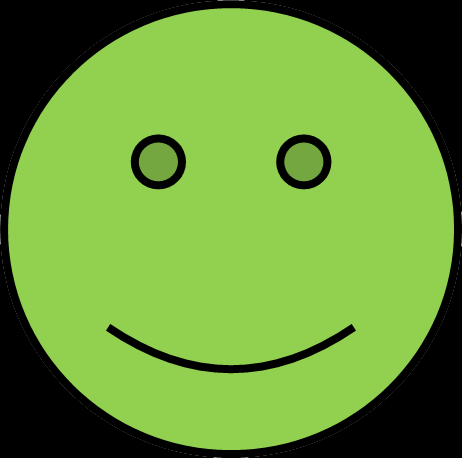


*Note.* This figure shows an example of the task interface (top panel) and stimuli used during each task (bottom panel).
