# Supplementary material for: Effects of pay rate and instructions on attrition in crowdsourcing research
Source: PLoS One. 2023 Oct 4;18(10):e0292372. doi: 10.1371/journal.pone.0292372 (PMC10550147; doi:10.1371/journal.pone.0292372)
Supplement: Attachment — Submitted filename: Response to Reviewers_7.3.23.docx [file pone.0292372.s002.docx]

The Abstract is clear but could benefit from the inclusion of the actual sample size employed in the study.

**We added this information to the abstract.**

The first sentence of the manuscript is unclear as to whether the authors are asserting that half of all participants for research in psychology and social sciences are recruited through crowdsourcing, or if half of all research studies in psychology and social sciences use crowdsourcing to recruit participants.

**The latter is correct. We have changed the first sentence to improve clarity.**

Related to this, is there any time frame as to the assertion underlying this claim given that crowdsourcing is relatively recent compared to studies undertaken in psychology and social sciences since the turn of the last century? Clearly there is a trend to use crowdsourcing but when did this generally commence?

**This began a little over a decade ago, we added this information to the first sentence (e.g., http://journal.sjdm.org/10/10630a/jdm10630a.pdf)**

On page 9 and elsewhere, it is claimed that the findings suggest that performance accuracy increases in simple reversal learning tasks with increased pay rates, and that data quality was assessed. There is a need to include more information regarding the definition and criteria used by the authors to assess both accuracy and data quality. Was it simply based on obtained scores on these tasks, and could other factors be excluded from influencing such performance (e.g., subsample characteristics given we do not have data on age and sex distribution)?

**We added definitions for both. Regarding the last point, we mention on page 12 that one limitation is that demographic variables (e.g., age, sex) were not controlled for or assessed when examining performance accuracy.**

On page 11, ‘…hourly pay with ranging between…” should read ‘…hourly pay with ranging between…”.

**We changed this to “hourly pay ranging between…”**

The authors do not provide any information regarding the qualifications of the Prolific participants. When constructing a Prolific study, researchers are permitted the chance to “pre-screen” potential participants. A common approach is to limit recruitment to participants from certain countries and who have completed a minimum number of surveys already (e.g., 100) with a minimum approval rating. Perhaps the researchers used such screenings but failed to report them? If no prescreening was employed, a strong argument should be made as to why they felt these were unnecessary. From personal experience, data are substantially improved (along with attrition) when recruiting participants with established experience in the platform.

**We did not use prescreening because research suggests that, unlike MTurk, Prolific generally provides high-quality data without prescreening (Peer et al., 2021). We now include this information in the Methods section.**

In addition to minimum qualifications, it is common practice to use some form of quality control checking to ensure data are indeed valid. One tactic we have used in our research is to code qualitative data, of which the researchers have several potential questions. In my casual review, there are a sizable number of participants who did not answer any qualitative questions, which raises some skepticism as to the quality of their responses. I would advise the researchers to provide some form of systematic screening of the qualitative responses to rule out the potential for any inattentive data. It appears the researchers informed participants to “Leave the question blank if you prefer not to answer,” which complicates such an analysis. Regardless, I advise some screening process of some kind.

**We did thoroughly screen qualitative data – for example, we looked for nonsensical response patterns or responses that were not in English. However, we did not exclude any data sets for this reason. We added a note about this process to supplemental survey data. Unlike MTurk, Prolific takes a number of steps to eliminate bots (see** [**https://www.prolific.co/blog/bots-and-data-quality-on-crowdsourcing-platforms**](https://www.prolific.co/blog/bots-and-data-quality-on-crowdsourcing-platforms)**).**

Related to point #3 above, there seems to be many missed opportunities for additional analyses. For example, I ran a quick qualitative analysis on the data and found an interesting pattern in responses to the question: “What do you think was the overall purpose of the study you just completed?” On the average, high pay participants responded with about 30 total characters, while low pay participants responded with about 20 total characters. However, this was not statistically significant. I also found that high pay participants were less likely to put an “I don’t know” response—but again, this isn’t statistically significant.

**We appreciate this point and agree that there are many possible measures of data quality. We added a footnote with the suggestion to use number of characters on specific survey questions as a measure of data quality – see page 10.**

The question “What gender/sex do you identify with?” contains only 3 different responses: Male, Female, or Other. Restricting participants to essentially a binary choice does not seem like an inclusive survey, which could impact participant responses to other questions, or perhaps even their attrition.

**Thank you for this important suggestion. We agree and will modify this questionnaire for future research.**

Regarding attrition, it seems possible that some participants may not have completed the study because it required installing the Inquisit plug-in. It is thereby possible that some variance could be explained by this artifact. To better isolate contributing variables, future studies might consider keeping all tasks within one survey and avoiding the use of plug-in installs.

**We also appreciate this point and have added this as a limitation in the Discussion.**

Considering this study included pay amount as a primary IV, it is curious that the researchers did not include an income question in their demographics.

**We suggest that future researchers should evaluate how demographic variables influence the relation between pay rate and performance accuracy on Page 12, and we now mention income as another demographic variable that could be evaluated in that section.**

It appears that Prolific IDs may be present in the spreadsheet. I advise against sharing this information.

**These have been removed.**
